# *N*′-(3,5-Dichloro-2-hy­droxy­benzyl­idene)-4-(dimethyl­amino)­benzohydrazide methanol monosolvate

**DOI:** 10.1107/S1600536812010148

**Published:** 2012-03-10

**Authors:** Huanzhi Xu, Jingya Sun

**Affiliations:** a College of Marine Sciences, Zhejiang Ocean University, Zhoushan 316000, People’s Republic of China

## Abstract

The title compound, C_16_H_15_Cl_2_N_3_O_2_·CH_3_OH, a Schiff base molecule, is prepared by the reaction of 3,5-dichloro­salicyl­aldehyde with 4-dimethyl­amino­benzohydrazide in methanol. The Schiff base mol­ecule is approximately planar, with a mean deviation from the least-squares plane defined by the non-H atoms of 0.0452 (3) Å, and with a dihedral angle between the benzene rings of 4.2 (3)°. This planarity is assisted by the formation of an intra­molecular O—H⋯N hydrogen bond. In the crystal, adjacent Schiff base mol­ecules are linked by two methanol mol­ecules through N—H⋯O and O—H⋯O hydrogen bonds, forming dimers.

## Related literature

For the preparation of Schiff base compounds by the condensation reaction between aldehydes with organic primary amines, see: Miura *et al.* (2009 ▶); Zhao *et al.* (2010 ▶); Karadağ *et al.* (2011) ▶; Bingöl Alpaslan *et al.* (2010 ▶). For standard bond lengths, see: Allen *et al.* (1987 ▶).
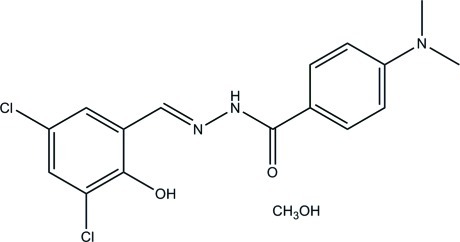


## Experimental

### 

#### Crystal data

C_16_H_15_Cl_2_N_3_O_2_·CH_4_O
*M* *_r_* = 384.25
Monoclinic, 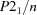

*a* = 7.6498 (15) Å
*b* = 14.338 (3) Å
*c* = 16.884 (2) Å
β = 103.076 (2)°
*V* = 1803.8 (5) Å^3^
*Z* = 4
Mo *K*α radiation
μ = 0.38 mm^−1^
*T* = 298 K
0.13 × 0.12 × 0.10 mm

#### Data collection

Bruker SMART CCD area-detector diffractometer
Absorption correction: multi-scan (*SADABS*; Bruker, 2001 ▶) *T*_min_ = 0.952, *T*_max_ = 0.963
8311 measured reflections
3238 independent reflections
1790 reflections with *I* > 2σ(*I*)
*R*_int_ = 0.072

#### Refinement

*R*[*F* ^2^ > 2σ(*F* ^2^)] = 0.079
*wR*(*F* ^2^) = 0.180
*S* = 1.03
3238 reflections
231 parameters
H-atom parameters constrained
Δρ_max_ = 0.24 e Å^−3^
Δρ_min_ = −0.22 e Å^−3^

### 

Data collection: *SMART* (Bruker, 2007 ▶); cell refinement: *SAINT* (Bruker, 2007 ▶); data reduction: *SAINT*; program(s) used to solve structure: *SHELXTL* (Sheldrick, 2008 ▶); program(s) used to refine structure: *SHELXTL*; molecular graphics: *SHELXTL*; software used to prepare material for publication: *SHELXTL*.

## Supplementary Material

Crystal structure: contains datablock(s) global, I. DOI: 10.1107/S1600536812010148/qm2057sup1.cif

Structure factors: contains datablock(s) I. DOI: 10.1107/S1600536812010148/qm2057Isup2.hkl

Supplementary material file. DOI: 10.1107/S1600536812010148/qm2057Isup3.cml

Additional supplementary materials:  crystallographic information; 3D view; checkCIF report

## Figures and Tables

**Table 1:** Hydrogen-bond geometry (Å, °)

*D*—H⋯*A*	*D*—H	H⋯*A*	*D*⋯*A*	*D*—H⋯*A*
N2—H2⋯O3^i^	0.86	2.10	2.848 (5)	145
O1—H1⋯N1	0.82	1.91	2.600 (5)	141
O3—H3⋯O2	0.82	1.97	2.771 (5)	166

Symmetry code: (i) 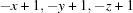.

**Fig. 1.**
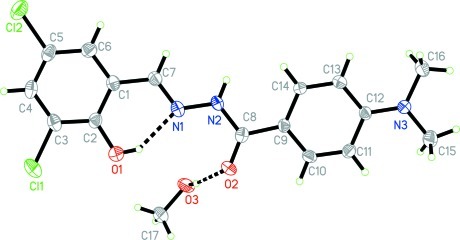
The molecular structure of the title compounds with atom labels and the 30% probability displacement ellipsoids. Hydrogen bonds are shown as dashed lines.

**Fig. 2.**
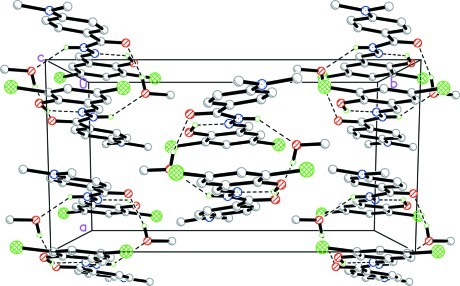
The molecular packing of the title compound. Hydrogen bonds are shown as dashed lines.

## supplementary crystallographic information

### Comment

The condensation reaction between aldehydes with organic primary amines readily forms Schiff bases containing the typical –C=N– groups (Miura *et al.*, 2009; Zhao *et al.*, 2010; Karadağ *et al.*, 2011; Bingöl Alpaslan *et al.*, 2010). In this paper, the new title compound (Fig. 1), was prepared by the reaction of 3,5-dichlorosalicylaldehyde with 4-dimethylaminobenzohydrazide in methanol.

The asymmetric unit comprises a Schiff base molecule and a methanol molecule. The Schiff base molecule is approximately planar, with mean deviation from the least squares plane defined by the non-hydrogen atoms of 0.0452 (3) Å, and with the dihedral angle between the two benzene rings of 4.2 (3)°. This planarity is assisted by the formation of an intramolecular O1—H1···N1 hydrogen bond (Table 1). All the bond lengths are within normal ranges (Allen *et al.*, 1987). In the crystal structure adjacent Schiff base molecules are linked by two methanol molecules through intermolecular N—H···O and O—H···O hydrogen bonds (Table 1) to form a dimer (Fig. 2).

### Experimental

3,5-Dichlorosalicylaldehyde (1.0 mmol, 0.190 g) and 4-dimethylaminobenzohydrazide (1.0 mmol, 0.179 g) were refluxed for 30 min in 30 ml me thanol, and cooled to room temperature to give colorless solid, which was isoloated by filtration. Single crystals of the title compound were formed by recrystallization of the solid product in methanol.

### Refinement

Hydrogen atoms were positioned geometrically and refined using the riding-model approximation, with C—H = 0.93–0.96 Å, O—H = 0.82 Å, N—H = 0.86 Å, and *U*_iso_(H) = 1.2*U*_eq_(C,*N*) or *U*_iso_(H) = 1.5*U*_eq_(O).

### Crystal data

**Table:**

C_16_H_15_Cl_2_N_3_O_2_·CH_4_O	*F*(000) = 800
*M**_r_* = 384.25	*D*_x_ = 1.415 Mg m^−^^3^
Monoclinic, *P*2_1_/*n*	Mo *K*α radiation, λ = 0.71073 Å
*a* = 7.6498 (15) Å	Cell parameters from 2546 reflections
*b* = 14.338 (3) Å	θ = 2.5–28.3°
*c* = 16.884 (2) Å	µ = 0.38 mm^−^^1^
β = 103.076 (2)°	*T* = 298 K
*V* = 1803.8 (5) Å^3^	Block, colorless
*Z* = 4	0.13 × 0.12 × 0.10 mm

### Data collection

**Table:**

Bruker SMART CCD area-detector diffractometer	3238 independent reflections
Radiation source: fine-focus sealed tube	1790 reflections with *I* > 2σ(*I*)
Graphite monochromator	*R*_int_ = 0.072
ω scans	θ_max_ = 25.5°, θ_min_ = 2.5°
Absorption correction: multi-scan (*SADABS*; Bruker, 2001)	*h* = −9→9
*T*_min_ = 0.952, *T*_max_ = 0.963	*k* = −16→17
8311 measured reflections	*l* = −13→20

### Refinement

**Table:**

Refinement on *F*^2^	Primary atom site location: structure-invariant direct methods
Least-squares matrix: full	Secondary atom site location: difference Fourier map
*R*[*F*^2^ > 2σ(*F*^2^)] = 0.079	Hydrogen site location: inferred from neighbouring sites
*wR*(*F*^2^) = 0.180	H-atom parameters constrained
*S* = 1.03	*w* = 1/[σ^2^(*F*_o_^2^) + (0.0664*P*)^2^ + 0.8315*P*] where *P* = (*F*_o_^2^ + 2*F*_c_^2^)/3
3238 reflections	(Δ/σ)_max_ < 0.001
231 parameters	Δρ_max_ = 0.24 e Å^−^^3^
0 restraints	Δρ_min_ = −0.22 e Å^−^^3^

### Special details

**Table:**

Geometry. All e.s.d.'s (except the e.s.d. in the dihedral angle between two l.s. planes) are estimated using the full covariance matrix. The cell e.s.d.'s are taken into account individually in the estimation of e.s.d.'s in distances, angles and torsion angles; correlations between e.s.d.'s in cell parameters are only used when they are defined by crystal symmetry. An approximate (isotropic) treatment of cell e.s.d.'s is used for estimating e.s.d.'s involving l.s. planes.
Refinement. Refinement of *F*^2^ against ALL reflections. The weighted *R*-factor *wR* and goodness of fit *S* are based on *F*^2^, conventional *R*-factors *R* are based on *F*, with *F* set to zero for negative *F*^2^. The threshold expression of *F*^2^ > σ(*F*^2^) is used only for calculating *R*-factors(gt) *etc*. and is not relevant to the choice of reflections for refinement. *R*-factors based on *F*^2^ are statistically about twice as large as those based on *F*, and *R*- factors based on ALL data will be even larger.

### Fractional atomic coordinates and isotropic or equivalent isotropic displacement parameters (Å^2^)

**Table:**

	*x*	*y*	*z*	*U*_iso_*/*U*_eq_	
Cl1	0.4325 (2)	0.29209 (10)	0.15198 (9)	0.0674 (5)	
Cl2	0.4175 (2)	0.65692 (11)	0.07222 (9)	0.0719 (5)	
N1	0.2957 (5)	0.4874 (3)	0.4015 (2)	0.0450 (10)	
N2	0.2686 (5)	0.5167 (3)	0.4749 (2)	0.0431 (10)	
H2	0.2702	0.5751	0.4864	0.052*	
N3	0.1064 (6)	0.5808 (3)	0.8296 (2)	0.0490 (11)	
O1	0.3448 (5)	0.3581 (2)	0.3007 (2)	0.0564 (10)	
H1	0.3013	0.3780	0.3376	0.085*	
O2	0.2453 (5)	0.3677 (2)	0.5161 (2)	0.0530 (10)	
O3	0.5727 (5)	0.3035 (2)	0.4941 (2)	0.0608 (10)	
H3	0.4685	0.3145	0.4960	0.091*	
C1	0.3425 (6)	0.5229 (3)	0.2711 (3)	0.0394 (12)	
C2	0.3621 (6)	0.4282 (3)	0.2505 (3)	0.0420 (12)	
C3	0.4009 (6)	0.4078 (4)	0.1756 (3)	0.0483 (13)	
C4	0.4178 (6)	0.4767 (4)	0.1208 (3)	0.0463 (13)	
H4	0.4441	0.4616	0.0712	0.056*	
C5	0.3950 (6)	0.5682 (4)	0.1411 (3)	0.0481 (13)	
C6	0.3577 (6)	0.5909 (4)	0.2145 (3)	0.0474 (13)	
H6	0.3422	0.6532	0.2266	0.057*	
C7	0.3097 (6)	0.5495 (3)	0.3482 (3)	0.0407 (12)	
H7	0.2984	0.6123	0.3599	0.049*	
C8	0.2386 (6)	0.4510 (4)	0.5302 (3)	0.0388 (12)	
C9	0.1976 (6)	0.4892 (3)	0.6054 (3)	0.0363 (11)	
C10	0.1902 (6)	0.4268 (3)	0.6673 (3)	0.0408 (12)	
H10	0.2087	0.3637	0.6592	0.049*	
C11	0.1562 (6)	0.4551 (3)	0.7403 (3)	0.0436 (12)	
H11	0.1497	0.4109	0.7799	0.052*	
C12	0.1311 (6)	0.5505 (3)	0.7556 (3)	0.0358 (11)	
C13	0.1359 (6)	0.6129 (3)	0.6922 (3)	0.0455 (12)	
H13	0.1173	0.6761	0.6996	0.055*	
C14	0.1672 (6)	0.5830 (3)	0.6193 (3)	0.0427 (12)	
H14	0.1681	0.6263	0.5784	0.051*	
C15	0.0961 (8)	0.5155 (4)	0.8937 (3)	0.0577 (15)	
H15A	0.0012	0.4716	0.8743	0.087*	
H15B	0.0723	0.5487	0.9395	0.087*	
H15C	0.2080	0.4827	0.9098	0.087*	
C16	0.0752 (7)	0.6783 (4)	0.8461 (3)	0.0591 (15)	
H16A	0.1787	0.7145	0.8425	0.089*	
H16B	0.0538	0.6841	0.8997	0.089*	
H16C	−0.0274	0.7007	0.8069	0.089*	
C17	0.5794 (7)	0.2197 (4)	0.4506 (3)	0.0619 (16)	
H17A	0.7002	0.1964	0.4626	0.093*	
H17B	0.5411	0.2315	0.3934	0.093*	
H17C	0.5016	0.1743	0.4665	0.093*	

### Atomic displacement parameters (Å^2^)

**Table:**

	*U*^11^	*U*^22^	*U*^33^	*U*^12^	*U*^13^	*U*^23^
Cl1	0.0718 (10)	0.0608 (9)	0.0729 (10)	0.0044 (7)	0.0235 (8)	−0.0241 (8)
Cl2	0.0840 (11)	0.0797 (11)	0.0536 (9)	−0.0156 (9)	0.0192 (8)	0.0149 (8)
N1	0.047 (3)	0.048 (3)	0.040 (3)	0.000 (2)	0.011 (2)	−0.009 (2)
N2	0.057 (3)	0.039 (2)	0.033 (2)	−0.002 (2)	0.010 (2)	−0.0098 (19)
N3	0.071 (3)	0.037 (2)	0.046 (3)	−0.001 (2)	0.026 (2)	0.000 (2)
O1	0.078 (3)	0.045 (2)	0.048 (2)	0.0047 (19)	0.018 (2)	0.0026 (18)
O2	0.074 (2)	0.035 (2)	0.055 (2)	0.0019 (17)	0.0234 (19)	−0.0095 (17)
O3	0.069 (2)	0.041 (2)	0.077 (3)	−0.0039 (19)	0.025 (2)	−0.0124 (19)
C1	0.035 (3)	0.046 (3)	0.034 (3)	−0.002 (2)	0.001 (2)	−0.005 (2)
C2	0.043 (3)	0.049 (3)	0.031 (3)	−0.002 (2)	0.002 (2)	0.001 (2)
C3	0.042 (3)	0.058 (3)	0.047 (3)	−0.002 (2)	0.014 (2)	−0.013 (3)
C4	0.037 (3)	0.070 (4)	0.032 (3)	−0.002 (2)	0.007 (2)	0.000 (3)
C5	0.044 (3)	0.062 (4)	0.036 (3)	−0.016 (3)	0.004 (2)	0.006 (3)
C6	0.050 (3)	0.044 (3)	0.046 (3)	−0.008 (2)	0.005 (2)	0.000 (3)
C7	0.042 (3)	0.040 (3)	0.039 (3)	0.002 (2)	0.007 (2)	−0.004 (2)
C8	0.029 (3)	0.050 (3)	0.037 (3)	−0.003 (2)	0.008 (2)	0.000 (3)
C9	0.036 (3)	0.038 (3)	0.038 (3)	−0.002 (2)	0.015 (2)	−0.003 (2)
C10	0.040 (3)	0.032 (3)	0.053 (3)	0.002 (2)	0.015 (2)	−0.002 (2)
C11	0.047 (3)	0.043 (3)	0.043 (3)	0.005 (2)	0.016 (2)	0.008 (2)
C12	0.036 (3)	0.036 (3)	0.037 (3)	−0.005 (2)	0.013 (2)	−0.005 (2)
C13	0.055 (3)	0.033 (3)	0.052 (3)	0.000 (2)	0.020 (3)	−0.003 (3)
C14	0.058 (3)	0.032 (3)	0.043 (3)	0.004 (2)	0.021 (2)	0.005 (2)
C15	0.070 (4)	0.063 (4)	0.042 (3)	0.003 (3)	0.015 (3)	−0.001 (3)
C16	0.068 (4)	0.054 (3)	0.062 (4)	0.001 (3)	0.030 (3)	−0.011 (3)
C17	0.074 (4)	0.053 (4)	0.063 (4)	0.009 (3)	0.024 (3)	−0.009 (3)

### Geometric parameters (Å, º)

**Table:**

Cl1—C3	1.736 (5)	C6—H6	0.9300
Cl2—C5	1.758 (5)	C7—H7	0.9300
N1—C7	1.288 (6)	C8—C9	1.480 (6)
N1—N2	1.367 (5)	C9—C10	1.387 (6)
N2—C8	1.381 (6)	C9—C14	1.394 (6)
N2—H2	0.8600	C10—C11	1.378 (6)
N3—C12	1.374 (6)	C10—H10	0.9300
N3—C15	1.447 (6)	C11—C12	1.414 (6)
N3—C16	1.456 (6)	C11—H11	0.9300
O1—C2	1.342 (5)	C12—C13	1.402 (6)
O1—H1	0.8200	C13—C14	1.374 (6)
O2—C8	1.222 (5)	C13—H13	0.9300
O3—C17	1.415 (6)	C14—H14	0.9300
O3—H3	0.8200	C15—H15A	0.9600
C1—C6	1.388 (6)	C15—H15B	0.9600
C1—C2	1.417 (6)	C15—H15C	0.9600
C1—C7	1.433 (6)	C16—H16A	0.9600
C2—C3	1.393 (6)	C16—H16B	0.9600
C3—C4	1.379 (7)	C16—H16C	0.9600
C4—C5	1.378 (7)	C17—H17A	0.9600
C4—H4	0.9300	C17—H17B	0.9600
C5—C6	1.372 (7)	C17—H17C	0.9600
			
C7—N1—N2	118.3 (4)	C14—C9—C8	125.3 (4)
N1—N2—C8	119.1 (4)	C11—C10—C9	122.2 (4)
N1—N2—H2	120.5	C11—C10—H10	118.9
C8—N2—H2	120.5	C9—C10—H10	118.9
C12—N3—C15	121.1 (4)	C10—C11—C12	120.6 (4)
C12—N3—C16	122.6 (4)	C10—C11—H11	119.7
C15—N3—C16	116.1 (4)	C12—C11—H11	119.7
C2—O1—H1	109.5	N3—C12—C13	121.7 (4)
C17—O3—H3	109.5	N3—C12—C11	121.6 (4)
C6—C1—C2	118.3 (4)	C13—C12—C11	116.7 (4)
C6—C1—C7	119.8 (5)	C14—C13—C12	121.6 (4)
C2—C1—C7	121.9 (5)	C14—C13—H13	119.2
O1—C2—C3	119.2 (5)	C12—C13—H13	119.2
O1—C2—C1	122.1 (4)	C13—C14—C9	121.5 (5)
C3—C2—C1	118.7 (5)	C13—C14—H14	119.3
C4—C3—C2	122.0 (5)	C9—C14—H14	119.3
C4—C3—Cl1	119.4 (4)	N3—C15—H15A	109.5
C2—C3—Cl1	118.6 (4)	N3—C15—H15B	109.5
C5—C4—C3	118.6 (5)	H15A—C15—H15B	109.5
C5—C4—H4	120.7	N3—C15—H15C	109.5
C3—C4—H4	120.7	H15A—C15—H15C	109.5
C6—C5—C4	120.9 (5)	H15B—C15—H15C	109.5
C6—C5—Cl2	119.9 (4)	N3—C16—H16A	109.5
C4—C5—Cl2	119.2 (4)	N3—C16—H16B	109.5
C5—C6—C1	121.5 (5)	H16A—C16—H16B	109.5
C5—C6—H6	119.3	N3—C16—H16C	109.5
C1—C6—H6	119.3	H16A—C16—H16C	109.5
N1—C7—C1	120.7 (4)	H16B—C16—H16C	109.5
N1—C7—H7	119.7	O3—C17—H17A	109.5
C1—C7—H7	119.7	O3—C17—H17B	109.5
O2—C8—N2	120.9 (4)	H17A—C17—H17B	109.5
O2—C8—C9	123.7 (4)	O3—C17—H17C	109.5
N2—C8—C9	115.4 (4)	H17A—C17—H17C	109.5
C10—C9—C14	117.3 (4)	H17B—C17—H17C	109.5
C10—C9—C8	117.4 (4)		

### Hydrogen-bond geometry (Å, º)

**Table:**

*D*—H···*A*	*D*—H	H···*A*	*D*···*A*	*D*—H···*A*
N2—H2···O3^i^	0.86	2.10	2.848 (5)	145
O1—H1···N1	0.82	1.91	2.600 (5)	141
O3—H3···O2	0.82	1.97	2.771 (5)	166

Symmetry code: (i) −*x*+1, −*y*+1, −*z*+1.
